# Factor structure and psychometric properties of the Arabic version of the 9-item Trier Inventory for Chronic Stress (TICS-9)

**DOI:** 10.1371/journal.pone.0360750

**Published:** 2026-11-05

**Authors:** Salman Althobaiti, Raseel Obaid, Salman Alharbi, Ghizlan Aljumah, Rahaf Alsharif, Abdulaziz Aldubiani, Mansour Mahmoud, Bjarne Schmalbach, Katja Petrowski, Ahmad Assinnari

**Affiliations:** 1 Department of Family and Community Medicine and Medical Education, College of Medicine, Taibah University, Madinah, Saudi Arabia; 2 College of Medicine, Taibah University, Madinah, Saudi Arabia; 3 Department of English Language and Translation, Qassim University, Buraydah, Saudi Arabia; 4 Alrowad Private School, Riyadh, Saudi Arabia; 5 Clinical and Simulation Laboratory, College of Medicine, Taibah University, Madinah, Saudi Arabia; 6 Department of Pharmacy Practice, College of Pharmacy, Taibah University, Madinah, Saudi Arabia; 7 Medical Psychology and Medical Sociology, University Medical Center of the Johannes Gutenberg University, Mainz, Germany; 8 Department of Basic Medical Science, College of Medicine, Taibah University, Madinah, Saudi Arabia; 9 Health and Life Research Centre, Taibah University, Madinah, Saudi Arabia; ISSEP Kef: Universite de Jendouba Institut Superieur du Sport et de l’Education Physique du Kef, TUNISIA

## Abstract

**Background:**

Chronic stress is a major determinant of physical and psychological health, yet validated instruments for assessing long-term psychosocial stress remain limited in Arabic-speaking populations.

**Objective:**

The present study aimed to translate and culturally adapt the 9-item Trier Inventory for Chronic Stress (TICS-9) into Arabic and to evaluate its psychometric properties in an adult sample.

**Methods:**

The English 9-item short version of the TICS was translated following International Test Commission guidelines, including independent forward translations, back-translation, and expert committee review to ensure conceptual and linguistic equivalence. A cross-sectional online survey was conducted among 291 Arabic-speaking adults in Saudi Arabia. Internal consistency was assessed using Cronbach’s alpha and McDonald’s omega. Confirmatory factor analysis (CFA) was performed using robust weighted least squares estimation (WLSMV) appropriate for ordinal data. Construct-related evidence was explored using hierarchical regression analyses with demographic and medical variables.

**Results:**

The Arabic TICS-9 demonstrated excellent internal consistency (α = 0.876; ω = 0.904). CFA provided preliminary support for a unidimensional structure with moderate to strong standardized loadings (0.583–0.819). Model fit showed a mixed pattern in the initially specified model but improved after theoretically justified post hoc residual correlations were added between item pairs with overlapping content. Income was inversely associated with chronic stress scores after adjustment for education, work status, and marital status. Depression was associated with higher stress scores after controlling for other chronic conditions.

**Conclusion:**

These findings provide initial evidence of internal consistency and preliminary structural support for the Arabic TICS-9 in Saudi Arabic-speaking adults. Further studies are needed to evaluate test–retest reliability, convergent validity, and measurement invariance across populations.

## 1. Introduction

Chronic stress has been increasingly recognized as a critical determinant of physical and psychological health, with sustained exposure associated with a wide range of adverse outcomes [1,2]. Accurate and reliable assessment tools are therefore essential for identifying chronic stress experiences and advancing research in this field [2,3]. The Trier Inventory for Chronic Stress (TICS), developed by Schulz, Schlotz, and Becker, is a multidimensional self-report instrument designed to assess long-term stress exposure across multiple domains of everyday life [4,5]. It captures nine core areas of chronic psychosocial stress: work overload, social overload, pressure to perform, work discontent, excessive demands from work, lack of social recognition, social tensions, social isolation, and chronic worrying, which are considered central to the experience of prolonged stress [4,5]. Originally developed and validated in German and subsequently translated into English [6,7], the TICS-9 has demonstrated robust psychometric properties and has been widely employed in psychological and health-related research across different cultural contexts [4,5,8].

Although Arabic measures of perceived stress have previously been developed and evaluated, such as the Arabic version of the Perceived Stress Scale, these instruments primarily assess perceived stress rather than the multidimensional chronic stress domains captured by the TICS-9 [9]. To our knowledge, no Arabic version of the TICS-9 has previously undergone translation, cultural adaptation, and psychometric evaluation. Therefore, the present study addresses a specific measurement gap by translating and culturally adapting the TICS-9 into Arabic and examining its initial psychometric properties in Saudi Arabic-speaking adults, following established cross-cultural adaptation guidelines [10,11]. This work represents a necessary first step toward providing a linguistically accurate and culturally appropriate instrument for assessing chronic stress in Saudi Arabic-speaking adults and lays the foundation for subsequent investigations of the psychometric properties of the Arabic TICS-9.

## 2. Method

The TICS-9 was adapted into Arabic in line with the International Test Commission (ITC) guidelines for test translation and adaptation [10,11]. We used the 9-item short version of the Trier Inventory for Chronic Stress (TICS-9) [6], which is licensed under the Creative Commons Attribution 4.0 International License (CC BY 4.0); the original version of the scale is provided in S2 File. Prior to commencing the study, the publisher was contacted regarding reuse and translation of the instrument and confirmed that formal permission was not required provided that appropriate attribution to the original authors and source publication was maintained in accordance with the license terms. The TICS-9 was derived from the original Trier Inventory for Chronic Stress developed by Schulz, Schlotz, and Becker [4]. Participants are asked to indicate how often they experienced each situation during the previous three months using a five-point Likert scale ranging from 0 (never) to 4 (very often). Item scores are summed to obtain a total score ranging from 0 to 36, with higher scores indicating greater chronic stress. Previous studies reported good reliability and validity for both the German and English versions of the instrument [5,6].

Initially, three independent bilingual physicians (a physiologist, a community physician, and a general practitioner), all native Arabic speakers fluent in English, translated the items from English into Arabic. The translators worked independently. Their forward translations were systematically compared by the research team, and discrepancies in wording, meaning, and cultural appropriateness were discussed until consensus was achieved, resulting in a unified preliminary Arabic version.

This preliminary version was subsequently back-translated into English by two independent native Arabic-speaking bilingual experts: an English language teacher and a professional medical English translator. Both were blinded to the source English version of the TICS-9 [6]. The original and back-translated versions were then reviewed by an expert committee consisting of a clinical pharmacist and a medical academic with expertise in psychometric research. The committee evaluated semantic, conceptual, and linguistic equivalence between versions and resolved any remaining discrepancies by consensus. The final Arabic version retained all nine items of the original TICS-9. No items were added, removed, or substantively modified. The final Arabic version of the TICS-9 is provided as S3 File, subject to the applicable licensing and attribution requirements.

This study was approved by the Institutional Review Board of Labayh Health Company (Approval No. RC2508). The study was conducted in accordance with the Declaration of Helsinki. The Research Ethics Committee approved the electronic informed consent procedure used in this study. All participants provided electronic informed consent prior to participation. Participation was voluntary and anonymous. No personal identifiers, including names, telephone numbers, email addresses, or IP addresses, were collected or stored.

The target population consisted of Arabic-speaking adults aged 18 years or older residing in Saudi Arabia. Inclusion criteria were age ≥ 18 years, ability to read Arabic, residence in Saudi Arabia, internet access, and provision of informed consent. Exclusion criteria included failure of attention-check questions and incomplete responses.

Convenience sampling with snowball dissemination was employed. The survey link was distributed exclusively through WhatsApp and circulated through university-affiliated social groups across different regions of Saudi Arabia (started on 29/12/2025 and ended on 30/1/2026). A total of 323 participants submitted the survey. Because the survey link was disseminated through open social networks and forwarding was encouraged, the response rate could not be calculated. No financial or non-financial incentives were provided.

Besides the demographic questions, three attention-check questions consisting of simple arithmetic problems (e.g., “5 + 5 =?”) were embedded within the survey to identify random or inattentive responding. Participants who answered two or more of the three attention-check questions incorrectly were excluded from the final analysis. Based on this criterion, 32 respondents were excluded. The final sample size of 291 participants was close to commonly cited recommendations of approximately 300 participants for stable factor solutions and exceeded the minimum participant-to-item rule of thumb of at least 10 participants per item. In the SEM output, the initially specified CFA model estimated 45 free parameters, and the modified model estimated 47 free parameters, resulting in participant-to-parameter ratios of approximately 6.5:1 and 6.2:1, respectively. Given the nine-item structure of the TICS-9, the available sample was considered adequate for confirmatory factor analysis.

Data cleaning, recoding, and descriptive statistics were performed using the Statistical Package for the Social Sciences (IBM SPSS Statistics, Version 27.0; IBM Corp., Armonk, NY, USA). No items were reverse scored prior to calculating total TICS-9 scores. Consistent with previous validation studies of the TICS-9, confirmatory factor analysis (CFA) was selected a priori because the instrument possesses an established one-factor structure. CFA was performed by Structural equation modelling using Jamovi (Version 2.7.17, 2024) [Computer Software]. Items were treated as ordered categorical variables, and CFA was conducted using WLSMV estimation.

## 3. Results

### 3.1. Demographics

Out of 323 participants who filled in the form, only 291 were included in the final study based on their responses to the attention check questions. Participants were predominantly young adults (Mdn age 25 years, IQR: 21–50, Min 18, Max 69) recruited from Saudi Arabia through online convenience and snowball sampling. Demographic data can be found in Table 1. Self-reported chronic medical conditions were collected and used as exploratory construct-related evidence. Income categories were coded ordinally as 0 = < 3,000 SAR, 1 = 3,000–5,000 SAR, 2 = 5,000–10,000 SAR, and 3 = > 10,000 SAR. Summary of all medical chronicity collected from participants can be found in Table 2. These conditions were self-reported and coded 0/1.

**Table 1:** Summary of demographic factors.

Variable		Count	Column N %
Sex	Male	93	32.0%
	Female	198	68.0%
Education	Intermediate School	2	0.7%
	Secondary School	58	19.9%
	Diploma	32	11.0%
	Bachelor’s degree	171	58.8%
	Postgraduate	28	9.6%
Work	Unemployed	31	10.7%
	Student	137	47.1%
	Gov. Sector Employee	72	24.7%
	Private Sector Employee	35	12.0%
	Freelance Work	16	5.5%
Nationality	Non-Saudi	29	10.0%
	Saudi	262	90.0%
Marital status	Single	163	56.0%
	Married	108	37.1%
	Divorced	15	5.2%
	Widowed	5	1.7%
Income in SAR	<3k	135	46.4%
	3k-5k	30	10.3%
	5k-10k	38	13.1%
	>10k	88	30.2%

*No missing data.

**Table 2:** Summary of participants’ chronicity.

Condition	Count	Column N %
Diabetes	28	9.6%
Hypertension	36	12.4%
Cardiovascular Disease	11	3.8%
Hyperlipidaemia	38	13.1%
Chronic Kidney Disease	8	2.7%
Depression	21	7.2%
Anxiety	30	10.3%
Other Psychiatric diseases	17	5.8%

### 3.2. Reliability and confirmatory factor analysis

The internal consistency of the translated items was excellent (Cronbach’s alpha = 0.876; McDonald’s ω = 0.904). Item-total correlation matrix can be found in the supplementary materials (Table B in S1 File). Item-level floor and ceiling frequencies were examined using the proportions of participants selecting the minimum (0) and maximum (4) response categories. Several items demonstrated moderate floor or ceiling concentrations, particularly item 7 at the floor level and items 1, 6, and 9 at the ceiling level (Table A in S1 File). Upon using SEM for CFA, all nine items were loaded substantially onto a single latent factor (Table 3).

**Table 3:** Factor loadings from the initially specified one-factor CFA model of the Arabic TICS-9.

Latent factor	Observed item	Estimate	Standardized loading β
Chronic_Stress	First	1.000	0.804
	Second	0.912	0.733
	Third	0.779	0.627
	Fourth	0.725	0.583
	Fifth	0.863	0.694
	Sixth	0.925	0.743
	Seventh	0.806	0.648
	Eighth	0.943	0.758
	Ninth	1.019	0.819

Note. β represents the standardized factor loading from the initially specified one-factor CFA model.

The initially specified one-factor CFA model, estimated using DWLS/WLSMV for ordinal indicators, showed a mixed pattern of fit (Table 4). Scaled CFI and TLI indicated acceptable incremental fit, and SRMR was acceptable; however, scaled RMSEA was elevated. The initially specified model produced scaled χ^2^(27) = 153.2, p < .001, CFI = 0.948, TLI = 0.930, RMSEA = 0.127, 95% CI [0.108, 0.147], and SRMR = 0.047.

**Table 4:** Fit indices for the initially specified and modified one-factor CFA models.

Model	Scaled χ^2^(df)	p-value	CFI	TLI	RMSEA [95% CI]	SRMR
Initially specified one-factor model	153.2 (27)	<.001	0.948	0.930	0.127 [0.108, 0.147]	0.047
Modified one-factor model with correlated residuals between items 1–2 and 2–3	58.7 (25)	<.001	0.986	0.980	0.068 [0.046, 0.091]	0.030

Note. CFA = confirmatory factor analysis; WLSMV/DWLS = robust weighted least squares estimation for ordinal indicators; CFI = comparative fit index; TLI = Tucker–Lewis index; RMSEA = root mean square error of approximation; SRMR = standardized root mean square residual. Scaled fit indices are reported. The modified model included post hoc residual correlations between items 1–2 and 2–3 based on modification indices and theoretical content overlap.

Inspection of modification indices suggested residual correlations between items 1–2 and 2–3, both exceeding 10. These residual correlations were introduced post hoc and were retained only because they were theoretically interpretable. Items 1 and 2 both address perceptions of excessive demands and burden, whereas items 2 and 3 both reflect interpersonal obligations and difficulties arising from responsibilities toward others. Consequently, these item pairs may share variance beyond the common chronic stress factor.

The modified model, including correlated residuals between items 1–2 and 2–3, showed improved fit compared with the initially specified model: scaled χ^2^(25) = 58.7, p < .001, CFI = 0.986, TLI = 0.980, RMSEA = 0.068, 95% CI [0.046, 0.091], and SRMR = 0.030. Because the modified model was derived from inspection of modification indices and evaluated in the same sample, the improved fit should be interpreted as exploratory and requires confirmation in an independent sample. Therefore, the CFA findings should be interpreted as preliminary structural evidence rather than definitive confirmation of the one-factor structure.

### 3.3. Exploratory construct-related associations

These analyses were exploratory and were conducted to examine associations between TICS-9 scores and selected demographic and medical variables. They were not intended to establish convergent, discriminant, criterion-related, or known-groups validity. No formal correction for multiple comparisons was applied because these analyses were exploratory; therefore, the findings should be interpreted cautiously and require replication.

#### 3.3.1. Demographic factors.

A hierarchical multiple regression examined whether income was associated with total TICS-9 scores beyond education, work status, and marital status. Step 1 was not significant (R^2^ = .013, F(3, 287) = 1.25, p = .291). Adding income at Step 2 significantly improved model fit (ΔR^2^ = .015, ΔF(1, 286) = 4.27, p = .040), yielding R^2^ = .027. In the final model, income was the only significant predictor (B = −1.03, SE = 0.50, β = −0.16, t = −2.07, p = .040, 95% CI [−2.02, −0.05]), indicating lower chronic stress scores with higher income category (coded 0–3). Education, work status, and marital status were not significant (Ps > .05).

#### 3.3.2. Medical factors.

A hierarchical multiple regression tested whether self-reported depression – reported by only 21 participants (7.2%)– explained additional variance in total TICS-9 scores beyond other chronic conditions (diabetes, hypertension, cardiovascular disease, hyperlipidaemia, chronic kidney disease, anxiety, and other psychiatric disorders). Step 1 was not significant (R^2^ = .043, ΔF(7, 283) = 1.83, p = .081). Adding depression in Step 2 significantly improved model fit (ΔR^2^ = .017, ΔF(1, 282) = 4.98, p = .026), yielding a total R^2^ of.060. In the final model, depression was associated with higher total TICS-9 scores (B = 5.27, SE = 2.36, β = .163, t = 2.23, p = .026; 95% CI [0.62, 9.92]). This finding should be interpreted cautiously because only 21 participants (7.2%) reported depression.

## 4. Discussion

The present study aimed to translate and culturally adapt the Trier Inventory for Chronic Stress (TICS-9) into Arabic and to provide an initial evaluation of its psychometric properties in an Arabic-speaking adult sample. Overall, the findings indicate that the Arabic version of the TICS-9 demonstrated high internal consistency and preliminary structural support for assessing chronic psychosocial stress in the present Saudi Arabic-speaking adult sample [4,5].

The Arabic TICS-9 demonstrated excellent internal consistency, as reflected by high reliability coefficients (Cronbach’s α = 0.876; McDonald’s ω = 0.904). Item–total correlations were uniformly strong, suggesting that all items contributed meaningfully to the overall construct of chronic stress. These findings are consistent with previous validations of the German and English versions of the TICS-9 and support the internal coherence of the Arabic translation [5–8]. The item-level floor and ceiling concentrations observed for several items suggest that some response categories may be unevenly endorsed in community samples; future studies should examine whether this pattern is replicated in more diverse and clinical populations.

Confirmatory factor analysis provided preliminary structural evidence for the Arabic TICS-9. Results provided preliminary support for a unidimensional factor structure, with all nine items loading strongly onto a single latent chronic stress factor. Standardized factor loadings ranged from 0.583 to 0.819, indicating substantial associations between individual items and the underlying construct.

The initially specified one-factor model showed a mixed pattern of fit: CFI, TLI, and SRMR were acceptable, whereas RMSEA was elevated and should be interpreted as indicating residual model misfit rather than being dismissed solely on methodological grounds. Elevated RMSEA values have been reported in short unidimensional ordinal scales and may partly reflect model complexity and degrees of freedom rather than true model misspecification; however, this does not remove the need for cautious interpretation [12,13].

After inspection of modification indices, residual correlations were added between items 1–2 and 2–3. These modifications improved model fit and were theoretically interpretable because the relevant item pairs shared overlapping content related to excessive demands, burden, and interpersonal obligations. However, because these correlated residuals were introduced post hoc, the final model should be interpreted cautiously. Moreover, the modified model was developed and evaluated in the same sample; therefore, the improved model fit should be regarded as exploratory and requires confirmation in an independent sample. Taken together, the factor loadings and fit indices provide preliminary support for a unidimensional structure and align with the conceptualization of chronic stress as a global construct [4,5,8]. Yet, further CFA studies in larger and more representative Arabic-speaking samples are required before stronger conclusions can be drawn. Consequently, the present findings should be viewed as preliminary evidence supporting the proposed factor structure rather than definitive confirmation. The final interpretation is based on the modified model, which incorporated theoretically justified correlated residuals and demonstrated improved overall fit.

Exploratory associations with demographic and medical variables were examined to provide preliminary descriptive evidence, but these analyses were not intended to establish formal psychometric validity. Income emerged as a significant predictor of chronic stress scores, with lower income levels associated with higher TICS-9 scores, even after controlling for education, work status, and marital status. This finding is consistent with theoretical and empirical literature linking socioeconomic disadvantage to increased chronic stress exposure [1–3]. In addition, depression was significantly associated with higher chronic stress scores after controlling for other chronic medical and psychiatric conditions, which is in line with prior research demonstrating close links between chronic stress and depressive symptomatology [1,2,14]. These analyses were exploratory and should be interpreted cautiously, particularly given the absence of correction for multiple comparisons. Accordingly, the observed associations with income and depression should be regarded as preliminary construct-related findings that require replication in independent samples before stronger inferences can be made.

The development of an Arabic version of the TICS-9 addresses an important gap in the literature. Research on chronic stress in Arabic-speaking populations has largely relied on measures of perceived stress, or related stress constructs rather than multidomain measures of chronic stress exposure [9,15]. By capturing multiple domains of chronic stress within a single instrument, the Arabic TICS-9 allows for a more comprehensive assessment of long-term psychosocial stress and facilitates cross-cultural comparisons with studies using the original German and English versions [5–8].

Several strengths of the present study should be noted. The translation process followed internationally recognized guidelines, including multiple forward translations, independent back-translation, and expert committee review, enhancing the likelihood of semantic and conceptual equivalence between language versions [10,11]. Additionally, the sample size of 291 participants provided a sufficient basis for reliability estimation and confirmatory factor analysis [16,17].

Nevertheless, some limitations warrant consideration. The use of an online convenience sample may limit the generalizability of the findings, particularly to individuals with lower digital access or differing socioeconomic backgrounds. The sample was predominantly Saudi, female, highly educated, and included a substantial proportion of students and lower-income participants. Consequently, the findings should be interpreted as initial sample-specific evidence from Saudi Arabic-speaking adults, predominantly highly educated female, should not be taken as establishing population norms or universal psychometric properties for all Arabic-speaking populations until replicated in larger, more diverse, and independently recruited samples. Moreover, the cross-sectional design precludes assessment of test–retest reliability, temporal stability, and predictive validity. In addition, several chronic medical conditions were reported by relatively small numbers of participants, which may have reduced the stability and precision of the regression estimates obtained in the construct-related analyses. Further, formal regression diagnostic assessment was limited. Also, chronic conditions were self-reported, and some psychiatric variables (e.g., depression, anxiety, and other psychiatric disorders) may exhibit conceptual overlap, which should be considered when interpreting these findings. Furthermore, convergent and discriminant validity were not examined because established Arabic measures of related constructs, such as perceived stress, anxiety, or depression, were not administered concurrently in the present study. This represents a principal methodological limitation of the present study and means that the present findings should be regarded as initial psychometric evidence rather than comprehensive validation of the Arabic TICS-9. Future studies should examine test–retest reliability, convergent validity, discriminant validity, criterion-related validity, measurement invariance across demographic groups, and performance in clinical samples. The exploratory regression analyses reported in the present study should therefore be viewed as preliminary construct-related evidence and not as substitutes for convergent validity, discriminant validity, criterion-related validity, test–retest reliability, or measurement invariance testing.

## 5. Conclusion

This study addressed the absence of an Arabic version of the 9-item Trier Inventory for Chronic Stress by translating, culturally adapting, and conducting an initial psychometric evaluation of the Arabic TICS-9 in Saudi Arabic-speaking adults. The Arabic TICS-9 demonstrated high internal consistency and moderate to strong standardized factor loadings. The initially specified CFA model showed mixed fit, whereas the modified model with theoretically justified post hoc residual correlations showed improved fit; nevertheless, the findings should be interpreted cautiously because the modifications were data-driven.

These findings suggest that the Arabic TICS-9 may be useful for assessing chronic psychosocial stress in populations similar to the present sample, but they should not be generalized to all Arabic-speaking populations until replicated in larger and more representative samples. Nevertheless, the results should be interpreted cautiously because the study used online convenience sampling and did not examine convergent validity, discriminant validity, criterion-related validity, test–retest reliability, or measurement invariance. Further studies using larger and more representative Arabic-speaking samples are needed to confirm the factor structure, evaluate measurement invariance across demographic groups, examine temporal stability, and establish associations with related psychological and clinical constructs. The present study therefore represents an initial step toward the cross-cultural psychometric evaluation of the TICS-9 in Arabic rather than a complete validation of the instrument.

## Supporting information

S1 File Supplementary statistical materials. This file contains Table A, presenting item descriptive statistics including floor and ceiling frequencies, and Table B, presenting item-total statistics including the item-total correlation matrix.
(PDF)

S2 File The original scale. This file contains the original version of the scale used in the study.
(PDF)

S3 File Final Arabic version of the TICS-9. This file contains the final Arabic translation of the TICS-9 used in the present study, provided subject to applicable copyright, licensing, and attribution requirements.
(PDF)
