# Supplementary material for: Factor structure and psychometric properties of the Arabic version of the 9-item Trier Inventory for Chronic Stress (TICS-9)
Source: PLoS One. 2026 Nov 5;21(11):e0360750. doi: 10.1371/journal.pone.0360750 (PMC13739217; doi:10.1371/journal.pone.0360750)
Supplement: Supplementary file 1 [file pone.0360750.s001.pdf]

Tables in the supplementary materials.

Table A. Item descriptive statistics

Item Statistics

	Mean	Std. Deviation	N	Floor (%) (Score = 0)	Ceiling (%) (Score = 4)
١. أشعر بأنني مثقل بالمهام التي علي تنفيذها	2.45	1.259	291	6.9%	28.2%
٢. أشعر أحيانا بأنني مثقل بمسؤولياتي تجاه الآخرين	2.23	1.251	291	10.7%	20.3%
٣. هناك مواقف اجد صعوبة ان أكون مجاملا في تلبية رغبات الآخرين	2.24	1.169	291	7.6%	16.8%
٤. يجب عليَّ الوفاء بمسؤوليات أعارضها بشدة	1.75	1.299	291	21.3%	13.1%
٥. على الرغم من الجهد الذي أقوم به، لا أستطيع إدارة مهامي بالشكل المناسب	1.74	1.342	291	21.3%	15.1%
٦. اشعر ان ادائي لا يقدر بما فيه الكفاية	2.14	1.395	291	16.8%	22.3%
٧. المشادات الكلامية التي أخوضها كثيرا ما تصبح نزاعات طويلة الأمد	1.43	1.351	291	32.3%	12.4%
٨. في بعض الأحيان لا تتوفر لي الفرصة للتعبير عن مخاوفي	2.09	1.388	291	17.5%	20.3%
٩. أحيانًا تسيطر علي همومي بشكل كامل	2.19	1.390	291	15.5%	23.7%

Table B. Item-total statistics

Item-Total Statistics

	Scale Mean if Item Deleted	Scale Variance if Item Deleted	Corrected Item-Total Correlation	Squared Multiple Correlation	Cronbach's Alpha if Item Deleted
١. أشعر بأنني مثقل بالمهام التي علي تنفيذها	15.79	56.532	.660	.553	.859
٢. أشعر أحيانا بأنني مثقل بمسؤولياتي تجاه الآخرين	16.00	57.734	.595	.493	.864
٣. هناك مواقف اجد صعوبة ان أكون مجاملا في تلبية رغبات الآخرين	16.00	59.317	.552	.338	.868
٤. يجب عليَّ الوفاء بمسؤوليات أعارضها بشدة	16.49	58.568	.522	.285	.871
٥. على الرغم من الجهد الذي أقوم به، لا أستطيع إدارة مهامي بالشكل المناسب	16.50	56.451	.614	.404	.863
٦. اشعر ان ادائي لا يقدر بما فيه الكفاية	16.10	55.028	.659	.448	.859
٧. المشادات الكلامية التي أخوضها كثيرا ما تصبح نزاعات طويلة الأمد	16.81	57.574	.548	.363	.869
٨. في بعض الأحيان لا تتوفر لي الفرصة للتعبير عن مخاوفي	16.15	54.867	.672	.485	.857
٩. أحيانًا تسيطر علي همومي بشكل كامل	16.05	53.925	.724	.544	.852
