# Supplementary material for: Factor structure and psychometric properties of the Arabic version of the 9-item Trier Inventory for Chronic Stress (TICS-9)
Source: PLoS One. 2026 Nov 5;21(11):e0360750. doi: 10.1371/journal.pone.0360750 (PMC13739217; doi:10.1371/journal.pone.0360750)
Supplement: Supplementary file 2 [file pone.0360750.s002.pdf]

Trier's Inventory for Chronic Stress (TICS-9)

- Answer based on your personal, subjective experience.
- Use the five-point Likert scale to rate each of the following items:

  0 = Never

  1 = Rarely

  2 = Sometimes

  3 = Often

  4 = Very Often

**1. I feel overwhelmed by my tasks**
**2. Sometimes I feel overburdened by my responsibilities toward others**
**3. I feel that my performance is not recognized enough!**
**4. In spite of the effort I make, I am unable to manage my tasks properly**
**5. I must meet responsibilities which I am adamantly opposed to**
**6. Sometimes I lack the opportunity to articulate my concerns!**
**7. There are situations in which I find it difficult to be obliging**
**8. Arguments I get involved in frequently become lasting conflicts**
**9. Sometimes I am consumed by my worries**
