# Supplementary material for: Factor structure and psychometric properties of the Arabic version of the 9-item Trier Inventory for Chronic Stress (TICS-9)
Source: PLoS One. 2026 Nov 5;21(11):e0360750. doi: 10.1371/journal.pone.0360750 (PMC13739217; doi:10.1371/journal.pone.0360750)
Supplement: Supplementary file 3 [file pone.0360750.s003.pdf]

Arabic version of the Trier's Inventory for Chronic Stress (TICS-9)

- أجب بناء على تجربتك الشخصية / الذاتية
- قيّم العبارات أدناه باستخدام أحد الكلمات التالية / الرجاء اختيار شدة موافقتك للعبارات أدناه باستخدام المقياس التالي

أبدا = 0

نادرا = 1

بعض الأحيان = 2

غالبا = 3

دائما = 4

١. أشعر بأنني مثقل بالمهام التي عليّ تنفيذها
٢. أشعر أحيانا بأنني مثقل بمسؤولياتي تجاه الآخرين
٣. أشعر ان ادائي لا يقدر بما فيه الكفاية
٤. على الرغم من الجهد الذي أقوم به، لا أستطيع إدارة مهامي بالشكل المناسب
٥. يجب عليَّ الوفاء بمسؤوليات أعارضها بشدة
٦. في بعض الأحيان لا تتوفر لي الفرصة للتعبير عن مخاوفي
٧. هناك مواقف أجد صعوبة ان أكون مجاملا في تلبية رغبات الآخرين
٨. المشادات الكلامية التي أخوضها كثيرا ما تصبح نزاعات طويلة الأمد
٩. أحيانًا تسيطر عليّ همومي بشكل كامل
